# e-health usage and health workers’ motivation and job satisfaction in Ghana

**DOI:** 10.1371/journal.pone.0239454

**Published:** 2020-09-23

**Authors:** Roger A. Atinga, Patience Aseweh Abor, Saratu Jenepha Suleman, Emmanuel Anongeba Anaba, Bii Kipo

**Affiliations:** 1 Department of Public Administration and Health Services Management, University of Ghana Business School, Legon, Accra, Ghana; 2 Department of Population, Family and Reproductive Health, University of Ghana School of Public Health, Legon, Accra, Ghana; Universiteit van Amsterdam, NETHERLANDS

## Abstract

**Background:**

The application of digital technology to improve health service delivery is increasing rapidly in Low- and Middle- Income Countries (LMICs). Digital tools such as electronic health (e-health) have been shown to improve healthcare quality, efficiency and patient satisfaction. However, evidence on health workers’ experiences using e-health services is limited in LMICs. This study examined the relationship between e-health usage and health workers’ motivation and job satisfaction.

**Methods:**

This was a cross-sectional survey design involving health workers across public and private hospitals in the Accra Metropolitan Assembly (AMA). A structured questionnaire was designed and self-administered to 305 respondents. Partial Least Square-Structural Equation Modelling (PLS-SEM) was employed to analyse the data.

**Results:**

Findings showed a significant positive association of job satisfaction with e-health (*p* < 0.01) and type of hospital (*p* < 0.01) but not motivation (p = 0.42). Although type of hospital significantly influenced job satisfaction (p < 0.01), it had no significant mediating effect on the relationship between e-health and job satisfaction. Finally, type of hospital interacted with e-health to moderate the association between e-health usage and job satisfaction.

**Conclusion:**

The findings suggest that e-health systems can catalyse health workers job satisfaction. Thus, measures to strengthen e-health structures to improve on their efficiency and effectiveness is crucial.

## Introduction

The application of Information Communication Technology (ICT) in healthcare is increasing rapidly in most LMICs [1,2]. In some of these countries, policies and programmes have been adopted ostensibly to promote and sustain the adoption and usage of e-health to facilitate healthcare delivery. For instance, in the quest to improve healthcare delivery and generate reliable, accurate and complete information for health policy interventions, Ghana’s Ministry of Health and its agencies are promoting the standard implementation of e-health systems. Ghana has implemented a national e-health strategy to promote the delivery of responsive, affordable and optimal healthcare as well as enhance communication and the use of information for planning, management and delivery of health services [3].

A range of definitions of e-health have been proffered [4–6]. However, there is seeming convergence of the definitions that e-health is 'the use of ICT or other digital technology to capture medical records, administer treatment, manage health and improve healthcare delivery’ [5]. The application of e-health revolves around public health, business and medical informatics provided through the internet or other technologies. e-health forms part of the series of innovations being introduced in healthcare in response to the quest to reduce cost and improve efficiency of health service delivery [7,8]. e-health is shown to have significant benefits. Its adoption results in improved patient experience, quality diagnosis and treatment, reliable prescription and quality clinical decision making [7,8]. e-health usage leads to timely access to health information, such as administrative and patients’ records, diagnosis and treatment profile records [9,10]. In addition, the use of ICT enables health workers to easily capture, store, retrieve, analyse and transmit large volumes of health information within and across care delivery points [11]. e-health has been found to be more cost-effective in promoting health education, self-management support and tele-monitoring in several domains of healthcare [7,12].

While interest for e-health has been driven primarily by the desire to improve clinical and administrative efficiency, these interests may not reflect those of health workers. Sustained use of e-health is largely dependent on the experiences of end-users such as health workers using it for various facets of daily patient care and management [13]. Studies attest that health workers who perceive e-health systems as easy to use are more likely to be satisfied with their jobs [14–16]. It has been established that physicians with higher perceived efficiency in the usage of e-health reported higher job satisfaction and commitment to care [17]. In addition, health workers motivated by e-health adoption are more likely to show continuous commitment to its usage. On the other hand, health workers are more likely to resist or discontinue e-health, if their experiences about its usage is poor. There is evidence that physicians who reported difficulties in using e-health also reported difficulties with clinical performance [18].

Health workers are key stakeholders in the design, implementation and usage of e-health. Thus, continuous use of e-health is linked to how it motivates and contributes to job satisfaction of health workers [19]. However, little is known about the extent to which health workers who use e-health to deliver healthcare are motivated and satisfied with their jobs. Arguably, little has been documented about e-health experiences among various cadres of health workers, especially in LMICs context. In Ghana, existing studies have focused on only physicians’ experience using e-health systems [19,20]. Limited attention has been given to whether e-health motivates and promotes job satisfaction among other clinical cadres such as nurses and allied health professionals who represent a larger proportion of users of e-health systems in health facilities. To the best of our knowledge, this is the first study in Ghana to examine the effect of e-health on health worker job satisfaction and motivation to care. Findings from this study will significantly contribute to the burgeoning literature on e-health and inform practice, research and interventions to enhance e-health adoption and acceptance in health service organisations.

## Methods

### Study design and sampling

Cross-sectional survey design was employed to collect data in hospitals located within the Accra Metropolitan Assembly (AMA). The metropolis has about 12 public and 28 private hospitals. At the time of the study, 5 public and 10 private hospitals had implemented digital systems including the application of cloud computing for e-health in all facets of service delivery arrangements–medical records, physician order entry, prescribing, decision support systems, cash flow management among others. The rest of the hospitals were either paper-based systems or hybrid (combination of paper and e-health systems). Simple random sampling was used to select 3 hospitals each of the population of public and private facilities with e-health systems.

For each hospital, doctors, nurses, pharmacists and laboratory (lab) technicians who routinely use e-health to perform clinical functions were sampled. The nurses were randomly selected such that at least 3 nurses from each department or unit participated in the study. We initially applied random selection of doctors, pharmacists and lab technicians in the hospitals. However, routine heavy workload owing to their few numbers severely constrained efforts to contact and interview doctors and the lab technicians on our random selection lists. This prompted us to use opportunistic sampling selection of those available and willing to participate in the study.

### Data collection methods

Data were collected using a questionnaire structured into 4 parts: background data, experience with e-health usage, and job satisfaction and motivation with the use of e-health. The background data included sex, age, profession, type of hospital and type of e-health system in use. Experience with the use of e-health, measured with 10 items asked respondents about their experiences using the e-health system for communicating diagnostic and treatment decisions, booking, prescribing, decision making support as well as the capture, storage and search of patient data. These questions were developed from a synthesis of the literature [21–23]. Based on the works of Boyer *et al*. [24] and Alharthi *et al*. [25], we measured job satisfaction using 6 items to solicit responses to whether the e-health system influenced respondents’ satisfaction with care delivery, commitment to work, clinical decisions and overall satisfaction with job. Motivation was measured with 4 items adopted from literature [26,27]. These questions asked respondents whether the e-health system motivates them to deliver quality services, work hard and achieve best therapeutic outcomes for patients. All the items were captured on a five-point Likert scale that ranged from 1 (strongly disagree) to 5 (strongly agree).

The questionnaire was pre-tested among 10 health workers in two hospitals within the AMA. Comments from the pre-test led to modification of the questionnaire by adding and dropping some items. Two of the authors SJS and BK led the data collection by visiting each hospital daily except weekends, to sample respondents and administer the questionnaire. Respondents completed the questionnaire at their own convenience during or after work except a few who completed and returned the tool at a different day. A total of 350 questionnaires were administered. However, 305 (a response rate of 81%) fully completed questionnaires were retrieved from the sampled nurses (140), doctors (58), pharmacists (51) and laboratory technicians (56). The study had the approval from Ghana Health Service.

### Data analysis

Data were analysed using Partial Least Square- Structural Equation Modelling (PLS-SEM) which allowed for estimating a network of relationship between the latent and measured constructs as well as maximising statistical power of the non-parametric data collected [28,29]. The use of PLS-SEM requires using key indicator model assessments such as indicator reliability, internal consistency, composite reliability and convergency validity [29]. Indicator reliability was assessed using confirmatory factor analysis to determine the proportion of variance of each indicator explained by the constructs. We loaded all the 16 indicators in the factor analysis. However, as seen in Table 1, only 12 indicators were strongly loaded above the ≥ 0.7 threshold [30] as follows: e-health usage (4), job satisfaction (5) and motivation (3). Thus, the constructs explained more than 50% of the indicators variance. To determine the internal consistencies of the indicators in measuring each construct, Cronbach Alpha (CA) coefficients were computed. The results from Table 1 show that all the CA were well above the recommended ≥ 0.7 ceiling [31]. This is indicative of good internal consistency of the indicators of each construct.

**Table 1:** Test results of standardised factor loadings and reliability.

Item	Item notation	Loading	CA	CR	AVE
**e-health usage**			0.955	0.968	0.882
The e-health system enables effective communication of clinical information to patients	EH2	0.892			
The e-health system enables effective capture and storage of patient data	EH4	0.963			
The e-health system facilitates clinical decision making	EH6	0.928			
The e-health system facilitates clinical information sharing across units and colleague clinicians	EH10	0.972			
**Job Satisfaction**			0.966	0.974	0.882
The e-health system has enhanced my commitment to work	SAT1	0.964			
I am satisfied using the e-health system to make clinical decisions	SAT2	0.973			
Generally, I am satisfied with the e-health system	SAT3	0.975			
With the e-health system, my performance at work is satisfactory	SAT4	0.946			
With the e-health system, I feel very satisfied with my clinical work	SAT5	0.830			
**Motivation**			0.956	0.874	0.701
I am happy with the e-health adoption at my workplace	MOT1	0.965			
The e-health system motivates me to work hard	MOT2	0.712			
With the e-health system, I am motivated to improve patient experience	MOT3	0.815			

CA: Cronbach Alpha; CR: Composite Reliability; AVE: Average Variance Extracted.

A disadvantage of CA coefficients is that the indicators are unweighted with the assumption that they are all equally reliable. To give relative weight to each indicator’s reliability, Composite Reliability (CR) values were computed. According to Jöreskog [32], satisfactory CR values must range between 0.70 and 0.9. As shown in Table 1, all the CR values were within acceptable range [33–34]. The constructs’ convergent validity was assessed by computing Average Variance Extracted (AVE) values. Satisfactory convergent validity is achieved when the AVE values are 0.5+ [30]. As shown in Table 1, the AVE values ranged from 0.701 to 0.882. This demonstrates that each construct explained more than 50% of its indicators’ variance.

**Fig 1:**
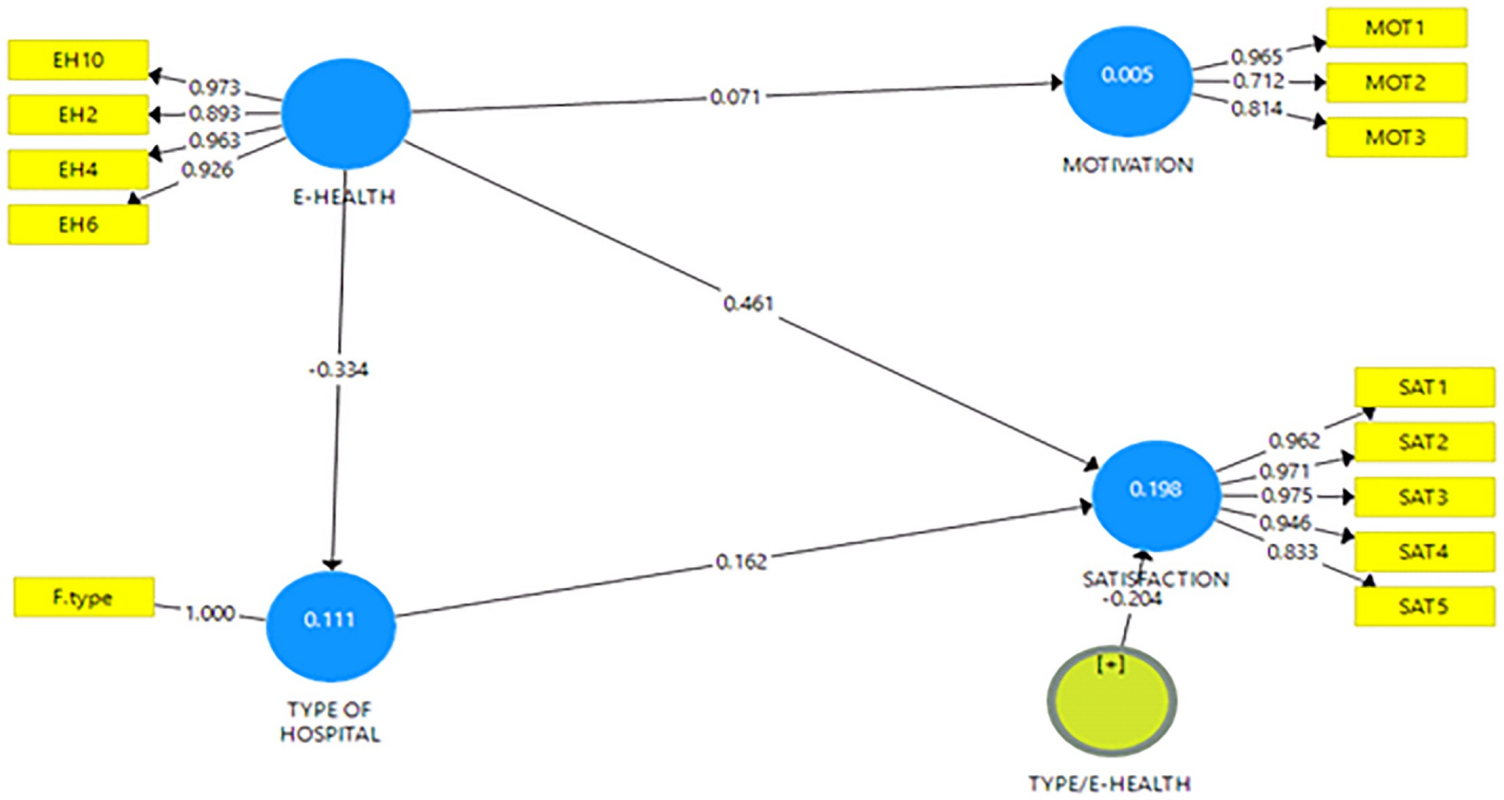
Tests statistics and path coefficients.

The associations between the dependent and independent variables were determine using path analysis. The path analysis was done by bootstrapping 5000 subsamples using two tailed tests (at α = 0.01). Positive and negative path coefficient demonstrates how variables influence each other in such directions. Additionally, the amount of variance in a path coefficient reflects the reliability of the influence [15]. The t-value associated with each of the standardised path coefficients is significant if p < 0.05 [13]. Arrows are used to illustrate the value-added paths that have significant influence (Fig 1).

## Results

### Descriptive statistics

The majority of the respondents were from public hospitals (59.7%), males (51.2%) and aged between 31–40 years (mean: 35; standard deviation: 29.6). The average job tenure was about 6 years (standard deviation: 9.1) (Table 2).

**Table 2:** Respondents’ characteristics, e-health type and type of hospital.

Socio-demographic variable	Frequency (n = 305)	Percent (%)
**Sex**		
Male	156	51.2
Female	149	48.8
**Age (in years)**		
≤ 30	125	41.0
31–40	147	48.2
41–50	33	10.8
**Years in service**		
1–5	177	38.4
6–10	119	39.0
11–20	67	22.6
**Type of hospital**		
Private	123	40.3
Public	182	59.7
**e-health type and functions**		
MP	27	8.9
COM	5	17.0
HRS	38	12.5
MP and CO	20	6.6
COM and HRDS	37	12.1
MP, COM, VID	21	6.9
MP, COM, HRDS	64	21.0
MP, COM, VID, HRDS	34	11.2
Others	12	3.8

MP: Mobile Phone, COM: Computer, HRDS: Health Related Digital System, VID: Videos.

### Path analysis

Results of the path coefficients are shown in Table 3. Findings showed a significant positive association of e-health with job satisfaction (p < 0.01) but not motivation (p = 0.42), demonstrating that health workers do not consider e-health system as an essential scheme of motivation at work. Further, the results revealed that type of hospital is significantly associated with job satisfaction (p < 0.001). Although e-health usage is positively associated with type of hospital, the type of hospital does not moderate the association between e-health usage and job satisfaction. Also, the type of hospital interacted with the type of e-health to moderate the relationship between e-health usage and job satisfaction. This suggests that the type of hospital more likely influences the type of e-health tools to be adopted, which in turn significantly influence job satisfaction of health workers. The path coefficients and t-values of the outer loadings are shown in Fig 1.

**Table 3:** Path coefficients of direct relationships and moderating effect of hospital type.

Path description	Path co-efficient	t-statistic	p-value
e-health usage -> Motivation	0.071	0.804	0.422
e-health usage -> Job satisfaction	0.461	8.095	0.000
Hospital type -> e-health usage	-0.334	6.298	0.000
Hospital type -> Job satisfaction	0.162	2.940	0.003
Hospital type × e-health usage -> Job satisfaction	-0.204	3.423	0.001

## Discussion

Electronic health records or e-health is evolving rapidly in developing countries [6]. This demonstrates a shift from the traditional paper-based system to digital technology application in healthcare delivery. While studies have shown the impact of e-health systems on better patient records management, improved diagnostic and treatment decisions and overall improvement in quality of care delivery [35–36], the extent to which e-health systems influence health workers job satisfaction and motivation to care remains less explicit. Accordingly, this study examined the associations between e-health usage and health workers motivation and job satisfaction in public and private hospitals.

The findings revealed statistically significant positive relationship between e-health usage and job satisfaction. Thus, a unit increase in e-health usage will lead to 46% increase in job satisfaction, holding other factors constant. The strength of the relationship was moderate. This finding suggests that health workers who used digital systems to communicate, register and consult patients experienced satisfaction with their job. Also, respondents who felt e-health enhances healthcare delivery were more likely to show job satisfaction. These findings correspond to related studies such as Botella *et al*. [37] who found an increase in job satisfaction and positive emotions among health workers after the adoption of e-health [38]. Jones *et al*. [16] also found that physicians who perceived that e-health systems are easy to use were more likely to be satisfied with their job. Similarly, Williams *et al*. [17] revealed that physicians with higher perceived efficiency to use e-health reported higher job satisfaction. This implies that to improve job satisfaction among health workers, healthcare managers should endeavour to connect, or ensure that e-health services have an impact on performance outcomes of health workers. In addition, healthcare managers must ensure that health workers have the requisite ICT skills and competencies to use e-health during the delivery of healthcare. This may require capacity building through training. Capacity building is important because clinicians who participate in e-health proficiency training are more likely to report an increase in job satisfaction and commitment to care [16].

Contrary to an existing study [11], findings showed no statistically significant relationship between e-health usage and motivation. The disparities in findings may be due to variations in contextual factors. In addition, the differences in findings can be attributed to the fact that health workers are motivated by extrinsic and intrinsic motivators. Too often health workers are motivated by extrinsic factors like financial incentives, better conditions of service among others [39]. In this regard, e-health, although an extrinsic issue may not be a strong motivating factor, since it does not directly yield reward or utility. Moreover, it has been shown that health workers consider their profession as a call to serve. For this reason, they may be motivated by intrinsic rewards, such as self-esteem, and not necessarily by extrinsic elements such as e-health usage [40,41]. Nonetheless, making e-health systems attractive, user friendly and incentivising continuous use of e-health for improved healthcare delivery can greatly spur up motivation and greater performance of health workers.

It was found that the type of hospital significantly influenced job satisfaction. This suggests that health workers in public and private hospitals with e-health systems experienced differential levels of job satisfaction. Previous studies confirmed a positive relationship between hospital type and job satisfaction among health workers. For instance, a study by Gudeta [42] found that private health workers in Ethiopia were more likely to be satisfied with their jobs compared to those in the public hospitals. Rana [43] conducted a cross-sectional study among health workers and established that private hospitals’ employees were more satisfied with their jobs than those in the public hospitals. By implication, health workers in private hospitals were more likely to show greater satisfaction with e-health usage than their counterparts in public hospitals. Prior studies also found different factors influencing job satisfaction among public and private hospitals health workers. Waqar and Hamid [44], for example, established that while health professionals in public hospitals showed increased job satisfaction due to reasonable working hours, relatively better wages and other benefits, that of private hospital workers attributed job satisfaction to positive feedbacks on performance, professional growth and promotion system. These findings suggest that factors that influence job satisfaction differ with regards to the type of hospital. Therefore, healthcare managers in private and public hospitals should consider complementing financial and non-financial incentives that increases satisfaction with the use of e-health services.

We found significant negative association between hospital type interaction with e-health and job satisfaction. This imply that the combine effect of hospital type and e-health inversely influence health workers job satisfaction. Overall, the findings indicate that although e-health is an innovative healthcare delivery tool, it may not be functionally beneficial to health workers in the public and private sectors. Going forward, healthcare managers will have to conduct need assessment before implementing digital systems. In addition, healthcare managers should ensure that the necessary structures are put in place prior to adopting digital systems. This will greatly boost motivation and satisfaction with usage of digital technologies.

### Limitations

Though this study provides valuable information for improving motivation and job satisfaction with e-health systems in Ghana, it is not without limitations. One limitation is our inability to recruit an equal number of participants from both private and public hospitals. Also, we conducted the study in an urban setting, which has different characteristics from rural settings. In this regard, interpretation of the findings must be done with caution. The cross-sectional nature and the small sample size of this study are also acknowledged as limitations.

## Conclusion

The adoption of electronic technology in healthcare delivery is increasing rapidly across the globe. However, evidence on the impact of e-health on job satisfaction and motivation of health workers is limited in developing countries like Ghana. The objective of this study was to investigate the relationship between e-health usage association with job satisfaction and motivation among health workers in the Accra Metropolitan Assembly. We hypothesised that e-health usage will have a positive influence on job satisfaction and motivation. However, we found that e-health usage had positive statistically significant influence on job satisfaction, but not motivation. Our study provides empirical evidence for scaling up electronic health services to improve health workers utility and motivation to care for patients.
